# Análisis de la variabilidad genética de una muestra de la población de Bogotá: hacia la constitución de un mapa de haplotipos

**DOI:** 10.7705/biomedica.4753

**Published:** 2019-09-01

**Authors:** Juan David Caicedo, Alejandro Cáceres, Carlos E. Arboleda-Bustos, María Fernanda Mahecha, Jenny Ortega, Gonzalo Arboleda, Humberto Arboleda

**Affiliations:** 1 Grupo de Neurociencias, Universidad Nacional de Colombia, Bogotá, D.C., Colombia Universidad Nacional de Colombia Grupo de Neurociencias Universidad Nacional de Colombia BogotáD.C Colombia; 2 Instituto de Salud Global de Barcelona, Barcelona, España Instituto de Salud Global de Barcelona Barcelona España

**Keywords:** proyecto mapa de haplotipos, proyecto 1.000 genomas, variabilidad genética, Colombia, HapMap Project, The 1000 Genome Project, genetic variability, Colombia

## Abstract

**Introducción.:**

Los proyectos del mapa de haplotipos (HapMap) y de los 1.000 genomas han sido fundamentales para la compresión del componente genético de las enfermedades comunes y los fenotipos normales. Sin embargo, la variabilidad genética colombiana incluida en estos proyectos no es representativa del país.

**Objetivo.:**

Contribuir al conocimiento de la variabilidad genética de la población colombiana a partir del estudio genómico de una muestra de individuos de Bogotá.

**Materiales y métodos.:**

Se genotipificaron 2’372.784 marcadores genéticos de 32 individuos nacidos en Bogotá y de padres originarios de la misma ciudad utilizando la plataforma Illumina™. Los niveles de variabilidad genética se determinaron y se compararon con los datos disponibles de otras poblaciones del proyecto de los 1.000 genomas.

**Resultados.:**

Los individuos analizados presentaron una variabilidad genética semejante a la de poblaciones con las que comparten ancestros. No obstante, a pesar de la poca diferenciación genética detectada en la población de Bogotá y en la de Medellín, el análisis de los componentes principales sugiere una composición genética diferente en las dos poblaciones.

**Conclusiones.:**

El análisis genómico de la muestra de Bogotá permitió detectar similitudes y diferencias con otras poblaciones americanas. El aumento de tamaño de la muestra bogotana y la inclusión de muestras de otras regiones del país permitirán una mejor compresión de la variabilidad genética en Colombia, lo cual es fundamental para los estudios de salud humana, y la prevención y el tratamiento de enfermedades comunes en el país.

## Abstract (en)

**Introduction:**

The HapMap and the 1000 Genomes projects have been important for understanding the genetic component of common diseases and normal phenotypes. However, the Colombian genetic variability included in these projects is not fully representative of our country.

**Objective:**

To contribute to the knowledge of the Colombian genetic variability through the genomic study of a sample of individuals from Bogotá.

**Materials and methods:**

A total of 2,372,784 genetic markers were genotyped in 32 individuals born in Bogotá whose parents are from the same region, using the Illumina™ platform. The genetic variability levels were determined and compared with the data available from other populations of the 1000 Genomes Project.

**Results:**

The genetic variability detected in the individuals from Bogotá was similar to those with shared ancestry. However, despite the low levels of genetic differentiation between Bogotá and Medellín, populations the principal component analysis suggested a different genetic composition in them.

**Conclusions:**

Our genomic analysis of a Bogotá sample allowed us to detect similarities and differences with other American populations. The increase of the Bogotá sample and the inclusion of samples from other regions of the country will improve our understanding of the genetic variability in Colombia, essential for studies of human health and the prevention and treatment of common diseases in our country.

Durante los últimos años, las técnicas de secuenciación masiva del ADN han permitido el análisis de millones de variantes genéticas en diferentes poblaciones humanas ^1^. Esta aproximación ha conducido a un aumento exponencial en la identificación de las causas genéticas de enfermedades raras y comunes, lo cual permitirá proyectar estos hallazgos al desarrollo de políticas de prevención y diagnóstico en un futuro muy próximo ^2^^-^^4^.

Los proyectos como el HapMap ^5^^-^^7^ y el de los 1.000 genomas ^8^^,^^9^ han servido de base para el diseño y el desarrollo de estudios de asociación entre millones de variantes genómicas y muchas enfermedades y fenotipos complejos. Dichos proyectos se han enfocado en la determinación de los patrones comunes de la variación genética del genoma humano por medio de la caracterización de la variabilidad genética, sus frecuencias y correlaciones en diferentes poblaciones humanas alrededor del mundo ^7^.

Sin embargo, a pesar de que la información existente en el proyecto de los 1.000 genomas incluye una muestra proveniente de individuos de Medellín ^9^, una región de Colombia que se caracteriza por una alta proporción de ascendencia caucásica ^10^, dicha variabilidad genética no es representativa de la población colombiana, cuya mayoría es mestiza y con un componente ancestral que proviene de poblaciones locales, europeas y africanas ^11^^,^^12^.

Por lo tanto, para tener una visión real de la variabilidad genética colombiana es necesario desplegar esfuerzos para hacer un registro más amplio e incluyente de los diferentes grupos y etnias de nuestro país. Este conocimiento permitirá el desarrollo de una medicina específica ajustada a las características genéticas de los colombianos.

En el presente estudio se analizó la variabilidad genética de una muestra de individuos mestizos de la población de Bogotá ^13^^,^^14^ y se comparó con los datos disponibles de diferentes poblaciones de los 1.000 genomas. Los hallazgos del estudio contribuyen al conocimiento de la variabilidad genética colombiana.

## Materiales y métodos

Selección de individuos

Se entrevistaron 32 individuos de ambos sexos y se elaboró su árbol familiar. Se estableció que todos ellos y sus padres habían nacido en Bogotá. Todos los participantes firmaron un consentimiento informado para participar en el estudio y autorizar la recolección de muestras de sangre periférica.

Genotipificación

El ADN genómico de cada individuo se extrajo de las muestras de sangre periférica con el estuche de purificación DNA Quick-gDNA MiniPrep™. Posteriormente, se genotipificaron 2’372.784 marcadores en cada individuo, empleando el Infinium Omni 2.5-8™, versión 1.3, Bead Chip de la plataforma Illumina™ ^15^.

Análisis de datos

Los archivos con las extensiones .map y .ped del formato PLINK ^16^, se obtuvieron tras el proceso de genotipificación y se convirtieron a formatos.bed, .bin y .fam para hacer los análisis posteriores empleando los siguientes paquetes de Bioconductor™ ^17^: el paquete snpStats se empleó para hacer el control de calidad seleccionando los polimorfismos de un solo nucleótido (*Single Nucleotide Polymorphisms,* SNP) con valores de *call rate* mayores de 0,8 y una frecuencia del alelo menor (*Minor Allele Frequency,* MAF) mayor de 0,01 ^18^^-^^20^. Este paquete también se empleó para determinar la posible desviación del equilibrio de Hardy-Weimberg de las variantes presentes en la población de estudio ^21^.

El análisis de los componentes principales se hizo con el paquete SNPRelate independientemente para la población bogotana de estudio y, posteriormente, se incluyeron los datos obtenidos del proyecto de los 1.000 genomas (9) en 14 poblaciones ubicadas en cuatro continentes de la siguiente forma: 1) América (AMR), incluidas las poblaciones de Puerto Rico (PUR), México (MXL) y colombianos de Medellín (CLM); 2) África (AFR), la cual incluye las poblaciones de americanos de ancestro africano (ASW), yorubas en Ibadan, Nigeria (YRI), y luhya en Webuye, Kenia (LWK); 3) Europa (EUR), incluidas las poblaciones de Finlandia (FIN), los americanos con ancestros del norte y el oeste de Europa (CEU), británicos en Inglaterra y Escocia (GBR), población ibérica en España (IBS) y toscana en Italia (TSI), y 4) Asia (AS), incluidas las poblaciones de chinos Han en Beijing (CHB), chinos Han del sur (CHS) y japoneses en Tokio (JPT).

El índice de fijación (parámetro estadístico *Fst)* se empleó para determinar el grado de diferenciación genética entre la población bogotana y el resto de las poblaciones analizadas.

## Resultados

**Figura 1:**
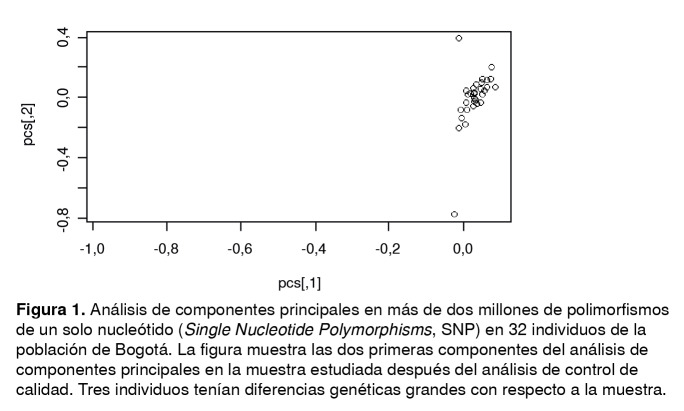
Análisis de componentes principales en más de dos millones de polimorfismos de un solo nucleótido (*Single Nucleotide Polymorphisms*, SNP) en 32 individuos de la población de Bogotá. La figura muestra las dos primeras componentes del análisis de componentes principales en la muestra estudiada después del análisis de control de calidad. Tres individuos tenían diferencias genéticas grandes con respecto a la muestra.

Se analizaron 32 individuos nacidos en Bogotá cuyos padres también habían nacido en la ciudad. En el análisis de los componentes principales de la población de estudio (figura 1), se observó que tres de los individuos presentaban una distancia genética más grande de la esperada con respecto al resto de la muestra debido, probablemente, a que sus generaciones anteriores tenían una ascendencia diferente a la bogotana y por ello se excluyeron de los análisis posteriores.

**Figura 2:**
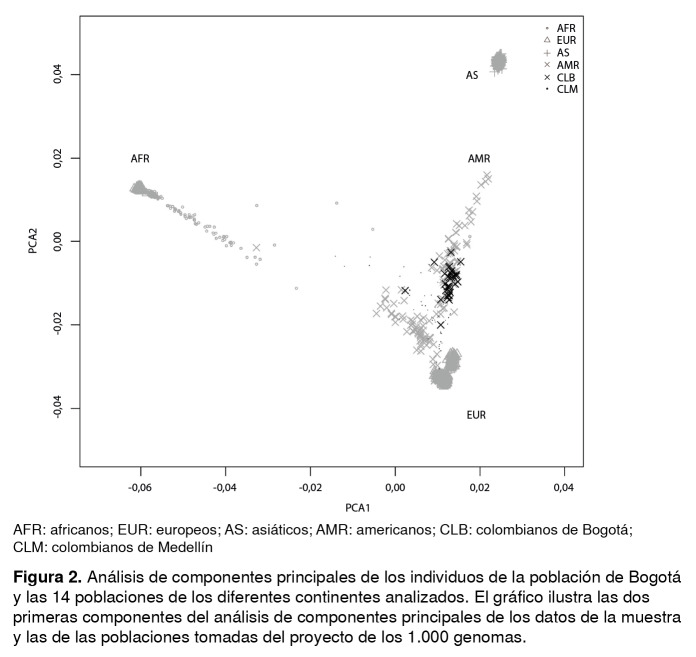
Análisis de componentes principales de los individuos de la población de Bogotá y las 14 poblaciones de los diferentes continentes analizados. El gráfico ilustra las dos primeras componentes del análisis de componentes principales de los datos de la muestra y las de las poblaciones tomadas del proyecto de los 1.000 genomas.

Los datos genómicos de 14 de las 26 poblaciones disponibles en el proyecto de los 1.000 genomas se emplearon con el propósito de compararlos con los datos de la población de estudio. En la figura 2 se observa que los individuos analizados tendieron a agruparse por continentes (AFR: africanos; AMR: americanos; EUR: europeos y AS: asiáticos).

No obstante, a pesar de que la población AMR se encuentra más cercana a la población EUR en comparación con las otras poblaciones, cabe destacar que la AMR presenta dos líneas genéticas claramente distinguibles: una línea más cercana a la población AS (amerindios), representada por la población mexicana (MXL), y la segunda, más cercana a la población AFR (afrodescendientes), representada por la población puertorriqueña (PUR).

Como era de esperarse, este análisis reveló que la variabilidad genética presente en la población bogotana de estudio (CLB) se ubicó dentro de la observada en la población AMR y fue muy similar a la de la población CLM. Sin embargo, esta última presenta una dispersión entre las dos líneas de la población AMR, en tanto que la población CLB se agrupó más homogéneamente dentro de la línea amerindia de la población AMR.

**Figura 3:**
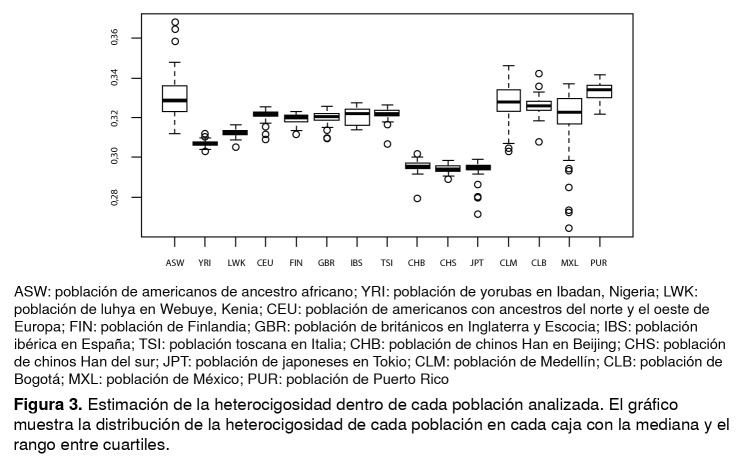
Estimación de la heterocigosidad dentro de cada población analizada. El gráfico muestra la distribución de la heterocigosidad de cada población en cada caja con la mediana y el rango entre cuartiles.

ASW: población de americanos de ancestro africano; YRI: población de yorubas en Ibadan, Nigeria; LWK: población de luhya en Webuye, Kenia; CEU: población de americanos con ancestros del norte y el oeste de Europa; FIN: población de Finlandia; GBR: población de británicos en Inglaterra y Escocia; IBS: población ibérica en España; TSI: población toscana en Italia; CHB: población de chinos Han en Beijing; CHS: población de chinos Han del sur; JPT: población de japoneses en Tokio; CLM: población de Medellín; MXL: población de México; PUR: población de Puerto Rico

El grado de diferenciación genética entre la población CLB y las diferentes poblaciones analizadas se determinó mediante el parámetro estadístico *Fst* (cuadro 1), el cual indicó que los niveles más bajos de diferenciación genética se dieron con las poblaciones CLM y MXL (*Fst* =0,003), en tanto que los más altos se detectaron con la población africana yoruba (YRI) (*Fst* =0,062). Por último, se detectaron diferencias en la variación de la heterocigosidad dentro de las poblaciones (figura 3). Por ejemplo, se observó una gran variación dentro de las poblaciones ASW, MEX y CLM, en tanto que otras poblaciones, como las YRI, LWK, CEU y JPT, presentan un índice notablemente bajo de variación genética. La población CLB presentó una variación genética intermedia.

**Cuadro 1:** Estimaciones de diferenciación genética (F*st*) entre la población bogotana (CLB) y el resto de las poblaciones analizadas

Comparación con la población bogotana (CLB)	Fst
ASW	0,041
YRI	0,062
LWK	0,055
CEU	0,012
FIN	0,012
GBR	0,012
IBS	0,013
TSI	0,012
CHB	0,029
CHS	0,030
JPT	0,031
CLM	0,003
CLM	0,003
PUR	0,008

ASW: población de americanos de ancestro africano; YRI: población de yorubas en Ibadan, Nigeria; LWK: población de luhya en Webuye, Kenia; CEU: población de americanos con ancestros del norte y el oeste de Europa; FIN: población de Finlandia; GBR: población de británicos en Inglaterra y Escocia; IBS: población ibérica en España; TSI: población toscana en Italia; CHB: población de chinos Han en Beijing; CHS: población de chinos Han del sur; JPT: población de japoneses en Tokio; CLM: población de Medellín; MXL: población de México; PUR: población de Puerto Rico

## Discusión

Este estudio de la variabilidad genética de 32 individuos de la población de Bogotá (CLB), reveló mayores similitudes genómicas con la población AMR que con las poblaciones AFR, EUR o AS (figura 2). A pesar de lo reducido de la muestra, fue suficiente para revelar posibles diferencias con poblaciones cercanas, especialmente con la población colombiana de Antioquia (CLM). Por ejemplo, a pesar de que la diferenciación genética entre las poblaciones CLB y CLM resultó baja (cuadro 1), los resultados obtenidos a partir de los análisis de análisis de componentes principales (figura 2) indicaron una composición genética diferente en estas dos poblaciones.

Estos hallazgos sugieren la presencia de posibles particularidades genéticas en cada población y, por lo tanto, la muestra de la población CLM de la base de datos de los 1.000 genomas no puede tomarse como representativa del país sino como una de sus subpoblaciones. Los futuros análisis con un mayor número de individuos de la población de Bogotá, así como de otras poblaciones del territorio colombiano, serán fundamentales para tener una mejor comprensión de la variabilidad genética de la población del país.

Por último, se pudo confirmar que los estudios de variabilidad genómica a partir de microarreglos (*microarrays*) son viables e informativos para caracterizar la genética de las poblaciones diversas y mestizas como las de Colombia. Los archivos obtenidos en este estudio estarán disponibles en la página web del Instituto de Genética de la Universidad Nacional de Colombia (www.genetica.unal.edu.co). El presente análisis piloto ofrece una base para desarrollar este tipo de estudios en el país.
